# Antioxidant and Immunostimulatory Activities of Fermented Sour Soybean Milk Added With Polypeptides From *Pleurotus eryngii*

**DOI:** 10.3389/fmicb.2022.750039

**Published:** 2022-06-15

**Authors:** Xinling Song, Ximin Xu, Wei Chen

**Affiliations:** ^1^College of Life Sciences, Shandong Agricultural University, Taian, China; ^2^College of Food Science and Engineering, Shandong Agricultural University, Taian, China

**Keywords:** *Pleurotus eryngii*, polypeptide, sour soybean milk, antioxidant, immunoregulation, intestinal flora

## Abstract

The improved quality of sour soybean milk by adding polypeptide from *Pleurotus eryngii* was investigated in this study, and the immunomodulatory effect of sour soybean milk fermented with polypeptides from *P. eryngii* was also evaluated in immunosuppressed mice induced by cyclophosphamide. Results showed the physicochemical property of sour soybean milk fermented with small-molecular-weight polypeptide (<3 kDa) were superior to the others including the decrease of pH, and increase of acidity, water-holding capacity and lactic acid bacteria count. The animal experiment demonstrated that sour soybean milk with polypeptide could effectively reverse the decreasing trend of thymus/spleen index and hematological parameters, enhance murine immune functions including serum hemolysin and splenic lymphocyte proliferation, and inhibit oxidative stress. In addition, sour soybean milk fermented with polypeptide could increase the diversity of intestinal flora, and increase the abundances of *Firmicutes*, *Bacteroides*, and *Lactobacillus*. Taken together, it could provide a theoretical basis for developing an immunomodulatory agent or functional food additives with antioxidant activity.

## Introduction

Soybean is one of the most important legume crops in the world, which contains abundant physiological active substances, including protein, phospholipid, sterol, oligosaccharides, dietary fiber, and so on (Liu et al., 2019; Hwang et al., 2021). Soybean milk is widely recognized as an alternative beverage of milk because it could alleviate abdominal discomfort symptoms such as abdominal pain and diarrhea caused by lactose intolerance (Li et al., 2020; Piazentin et al., 2020). However, some people may not prefer soybean milk because of its sensory characteristics similar to those of raw beans, and flatulence resulting from metabolization by the intestinal microbiota (Kim and Han, 2019). Studies have shown that fermentation is one of the main techniques used to increase nutritional qualities and improve flavor (Cai et al., 2021). In particular, sour soybean milk, a new kind of fermented product made by lactic acid bacteria, have attracted attention in food research due to their health values such as antioxidant, anticancer, anti-inflammatory, anti-diabetic and immunomodulatory effects as well as promote intestinal peristalsis (Bao and Chi, 2016; Yi et al., 2019; Li et al., 2020; Piazentin et al., 2020; Cai et al., 2021; Hwang et al., 2021). Moreover, fermented soybean milk can increase total amino acid content, and relieve diarrhea and constipation (Li et al., 2020; Cai et al., 2021). Therefore, it has broad market prospects to develop fermented sour soybean milk with appropriate acidity and good flavor.

Recently, increasing evidence has indicated that peptides derived from natural products especially mushrooms have been proven to possess excellent effects because of their low molecular weight, high activity, and easy absorption (Bhat et al., 2015; Arise et al., 2016; Kimatu et al., 2017; Sun et al., 2017). *Pleurotus eryngii*, known as the king oyster mushroom, has been used widely worldwide as both a food and a traditional mushroom attributed to its delicious taste and flavor as well as rich nutrients (Ren et al., 2016). Many studies have demonstrated that *P. eryngii* is suitable for low-calorie diets because it contains little fat and carbohydrates, and it has higher protein content than most mushrooms (Han et al., 2011; Sun and Li, 2017). Some of the bioactive polypeptides from *P. eryngii* have been reported to have a variety of health benefits such as antioxidant, anti-inflammation, antimicrobial, and immunoregulation activity (Wang and Ng, 2004; Han et al., 2011; Sun et al., 2017; Yuan et al., 2017; Hu et al., 2018). Hu et al. (2018) have isolated the bioactive *P. eryngii* polypeptide and reported it is the immunomodulatory protein that can boost cellular immune responses, and Yuan et al. (2017) have reported that a novel bioactive polypeptide was isolated from *P. eryngii* and it might be a good candidate for anti-inflammation in the gastrointestinal tract. All of these have revealed the potential for the application of *P. eryngii* bioactive polypeptides in the development of health foods and pharmaceutical products, thereby providing new insight as functional food supplements.

Therefore, this study was aimed to obtain sour soybean milk fermented with bioactive polypeptides from *P. eryngii* and evaluate the potential antioxidant and immunostimulatory activities, providing theoretical supports for the research and development of the bioactive peptides as the most suitable candidates in functional food.

## Materials and Methods

### Materials and Chemicals

Fruiting bodies of *P. eryngiis* were purchased from a local supermarket (Tai’an, China). The lactic acid bacteria starter was provided by Beijing Chuanxiu Technology Co., Ltd. (Beijing, China). Diagnostic kits for investigating the activities of superoxide dismutase (SOD), catalase (CAT), and total antioxidant capacity (T-AOC), as well as the contents of malondialdehyde (MDA) were purchased from Nanjing Jiancheng Bioengineering Institute (Nanjing, China). Cyclophosphamide (CTX) was provided by Shanghai XinYu Biotechnology Co., Ltd. (Shanghai, China). Culture medium RPMI-1640, sheep red blood cell (SRBC), red blood cell lysis buffer, trypan blue, and fetal calf serum were provided by Solarbio Science & Technology Co., Ltd. (Beijing, China). Cell counting kit-8 (CCK-8) was purchased from Beyotime Biotechnology Co., Ltd. (Shanghai, China). All other chemicals and reagents used in this experiment were from local chemical suppliers in China.

### Preparation of Polypeptides

The preparation of polypeptides from *P. eryngii* was performed according to the previous method described by He et al. (2013) and Kimatu et al. (2017) with some modifications. Briefly, the *P. eryngii* powder was added to 5% ice-cold acetic acid solution (v/v) containing 2-mercaptoethanol (0.1%, v/v) and stirred for 3 h at 4°C. The homogenate was centrifuged at 10,000 g for 20 min, and supernatants were filtered with Whatman filter paper No. 5, and ammonium sulfate was added to the cleared supernatant to 75% (w/v) saturation with precipitating for 12 h at 4°C. Then the mixture was centrifuged at 10,000 g for 20 min at 4°C, and the obtained precipitation was pooled, re-dissolved, and dialyzed at 4°C for 48 h against distilled water using 8,000–14,000 kDa dialysis membranes. The dialysate was lyophilized, collected, and stored at –20°C.

The *P. eryngii* proteins were dissolved into distilled water at the ratio of 1:25 (w/v) followed by stirring. Then the solution was adjusted to pH 9.0 before the addition of alcalase (5000 U/g, enzyme-substrate ratio) and hydrolyzed at 55°C for 4 h. The hydrolysates were immediately heated at 80°C for 20 min to stop the enzyme reaction. Subsequently, the hydrolysates were centrifuged at 8,000 g for 20 min at 4°C and the obtained supernatants were lyophilized, and the *P. eryngii* protein hydrolysate was named as PEPH.

The hydrolysate was separated by ultrafiltration membranes with cut-off molecular weight (Mw) of 1, 3, 5, and 10 kDa. The supernatant was first passed through the 1 kDa membrane and the retentate passed through 3 kDa, the 3 kDa retentate was passed through a 5 kDa membrane whose retentate was then passed through a 10 kDa membrane. The obtained fractions (5-10, 3-5, 1-3, and <1 kDa, named as PEP-10, PEP-5, PEP-3, and PEP-1, respectively) were concentrated by rotary evaporation (45°C), collected, lyophilized, and stored at –20 °C for further analysis.

### Assay of Antioxidant Activity

The antioxidant activities of the samples were expressed as IC_50_ (i.e., the amount of tested extract required for a 50% decrease in the absorbance of DPPH and hydroxyl radicals), while the reducing power was expressed as the values of absorbance.

The ability of the protein hydrolysate and polypeptide to scavenge the DPPH radical was determined according to the previous method with some modifications (Cho et al., 2014). In brief, 100 μL of DPPH (0.1 mmol/L) and 100 μL of aqueous polypeptide at various concentrations (0.2, 0.4, 0.6, 0.8, and 1.0 mg/mL) were mixed and reacted at room temperature for 30 min. The solution was mixed and the absorbance value was measured at 517 nm and ascorbic acid (vitamin C, Vc) solutions were used as control. The DPPH radical scavenging activity (DRSA) was calculated using the Equation (1):


(1) $$\text{DRSA}=\left[1-\left({\text{A}}_{\text{i}}-{\text{A}}_{\text{j}}\right)/{\text{A}}_{\text{o}}\right]\times 100\%$$

where A_0_ was the absorbance of the mixture including DPPH solution and ethyl alcohol, A_i_ was the absorbance of the mixture including samples and DPPH solution, and A_j_ was the absorbance of the mixture including samples and ethyl alcohol, respectively.

The ability of the protein hydrolysate and polypeptide to scavenge hydroxyl radical was determined referred with some modifications. Specifically, the reaction system containing 50 μL 1,10-Phenanthroline hydrate (2 mmol/L), 50 μL FeSO_4_ (2 mmol/L), 30 μL PBS (0.2 mol/L, pH 7.4), 20 μL H_2_O_2_ (30%, v/v) and 20 μL samples at various concentrations (0.2, 0.4, 0.6, 0.8, and 1.0 mg/mL) was incubated at 37°C for 60 min, and then the absorbance value was measured at 515 nm and Vc solutions were used as control. The hydroxyl radical scavenging activity (HRSA) was calculated by Equation (2):


(2) $$\text{HRSA}\left(\%\right)=\frac{{\text{A}}_{\text{S}}-{\text{A}}_{1}}{{\text{A}}_{0}-{\text{A}}_{1}}\times 100$$

where As was the absorbance value of the solution in the sample, A_1_ was the absorbance value of the solution in the absence of sample, A_0_ was the absorbance value of the solution in the absence of the sample and the reaction system.

The reducing power assay was carried out using the method reported by Sun et al. (2017). Briefly, the reducing power reagent was prepared by mixing phosphate buffer (0.2 mol/L, pH 6.6), 1% K_3_[Fe(CN)_6_], and 20 mmol/L trichloroacetic acids with the ratio of 10:1:1 (v/v/v). The mixture was incubated at 50°C for 20 min. Then 2.5 mL of the reducing power reagent was added to 1.0 mL of sample at various concentrations (0.2, 0.4, 0.6, 0.8, and 1.0 mg/mL) and incubated at 20°C for 30 min. The determination of reducing power was measured at 700 nm and Vc solutions were used as control.

### Preparation of Sour Soybean Milk

Soybeans were washed and soaked in water with 0.5% NaHCO_3_ for 12 h and ground, and then soybean mixture was strained through a 100-mesh sieve. 2% lactose, 6% saccharose, 0.3% PEP (polypeptides with molecular weight of <3 kDa) or PEPH and 0.5% gelatin were mixing, and 0.5% β-cyclodextrin was added at 60°C, then all heated to 90°C for 10 min. The mixture was sterilized at 100°C for 15 min and cooled until 25°C. Mixed lactic acid bacteria starters (including *Lactobacillus bulgaricus*, *Streptococcus thermophilus*, *Lactobacillus acidophilus*, *Lactobacillus plantarum*, and *Lactobacillus casei*) at the level of 0.5% were inoculated to soymilk and fermented at 42°C for 5.5 h, and ripen at 4°C for 12 h, the sour soybean milk fermented with PEP (polypeptides with a molecular weight of < 3 kDa) and PEPH were named as FPEP and FPEPH, respectively. The control sour soybean milk (SSM) was prepared by fermenting without polypeptides or *P. eryngii* protein hydrolysate.

### Physical and Chemical Index

The pH of samples was measured three times with a digital pH meter. The titratable acidity of the samples was determined according to the National Standard for Food Safety Determination of Acidity in Food (GB 5009.239-2016).

The lactic acid bacteria counts were counted according to the National Standard for Food Safety Determination of Lactic Acid Bacteria in Food (GB5009.5-2016), and presented as CFU/mL.

The water-holding capacity (WHC) of the sample was measured using the procedure of Abd El-Fattah et al. (2018) with slight modifications. 20 g fermented sour soybean milk was centrifuged at 5,000 g for 10 min, the whey in the supernate was weighted. WHC was calculated following the formula (3):


(3) $$\text{WHC}\left(\%\right)=\frac{{\text{m}}_{1}-{\text{m}}_{2}}{{\text{m}}_{1}}\times 100$$

where m_1_ was the weight of the fermented sour soybean milk, m_2_ was the weight of the whey.

The amino acid compositions of the samples were determined according to the National Standard for Food Safety Determination of Amino Acid in Food (GB 5009.124-2016) using an automatic amino acid analyzer (L-8900, Hitachi, Japan).

### Animal Experiment

The Kunming strain mice (SPF, male, 20 ± 2 g) were purchased from Pengyue Experimental Animal Breeding Co., Ltd [Production license number: SCXK (Lu) 20190003, Ji’nan, China]. The mice were housed under controlled conditions of 12 h light-dark cycles at 22 ± 2°C and 50–55% relative humidity with free access to water and standard food for 7 days. All the experiments were performed following the Regulations of Experimental Animal Administration issued by the State Committee of Science and Technology of the People’s Republic of China, and all mice procedures were reviewed and approved by the Animal Research Ethic Committee of Shandong Agricultural University, China.

After the 7-days acclimation period, all mice were randomly divided into ten groups including control group (NC), model group (MC), Vc group (Vc), sour soybean milk group (SSM), low-dosage (L-FPEP, 0.3%), middle-dosage (M-FPEP, 0.5%) and high-dosage (H-FPEP, 0.7%) small molecular weight polypeptide-supplemented groups, and low-dosage (L-FPEPH, 0.3%), middle-dosage (M-FPEPH, 0.5%) and high-dosage (H-FPEPH, 0.7%) PEPH-supplemented groups, respectively. The mice in the NC and MC groups were fed with saline (0.2 mL) once a day, the mice in the Vc group were fed with Vc solution (0.2 mL) every day, and the mice in the other groups were gavaged with corresponding sour soybean milk in the same amount during the consecutive 30-days. Five days before the end of the experiment, all mice except the NC group were intraperitoneally injected with cyclophosphamide 40 mg/kg body weight (Jie et al., 2020), while the NC group was injected with the same dosage of normal saline. Four days before the end, all mice were intraperitoneally injected with 0.2 mL 2% sheep red blood cell. Finally, all mice were fasted overnight and sacrificed under pathogen-free conditions abiding by the Principles.

Blood was drawn from their orbital veins using heparin as an anticoagulant for blood biochemical analysis. The white blood cell count, red blood cell count, lymphocyte, and hemoglobin levels were determined in the hematological examination. After centrifugation (6,000 r/min, 4 °C, 4 min) obtaining the serum, the activities of SOD, T-AOC, and CAT, and the content of MDA in serum were determined by the commercial reagent kits.

The fresh thymus and spleen were weighed to calculate the organ index (%) by formula (4).


(4) $$\mathrm{Organ}\mathrm{index}\left(\%\right)=\frac{m_{1}}{m_{2}}\times 100$$

where m_1_ was the thymus/spleen weights, and m_2_ was the final body weights.

### Serum Hemolysin Assay

The serum hemolysin was measured by Sun et al. (2011) and Jie et al. (2015) reported with slight modifications. Four days before the end of the experiment, all mice were intraperitoneally injected with 0.2 mL 2% sheep red blood cell. Finally, the mice were sacrificed for serum (2000 r/min, 10 min). Serum was diluted 100 times with saline water, then 1 mL serum, 10% SRBC suspension (0.5 mL, and 1 mL guinea pig serum) were added. By contrast, 1mL saline instead of mice serum was added to the blank tube. All tubes in 37°C water bath were heated for 30 min and then stopped by an ice water bath. After cooling, the tube was centrifuged at 2000 r/min for 10 min. The levels of serum hemolysin in all groups were measured at OD 540. 0.25 mL SRBC suspension (10%) was diluted to 4 mL and centrifuged at 2000 r/min for 10 min, the value of SRBC suspension was measured as SRBC_50_ at *OD*_540nm_. Determination of serum hemolysin was expressed as the half value of hemolysis (HC_50_) followed by the formula (5):


(5) $${\text{HC}}_{50}=\frac{OD_{1}-OD_{2}}{{\text{SRBC}}_{50}-OD_{2}}\times 100$$

where *OD*_1_ was the value of mice serum hemolysin, *OD*_2_ was the value of blank contrast, and SRBC_50_ was the value of SRBC suspension.

### Splenic Lymphocyte Proliferation Assay

The spleens of the mice were minced in culture medium RPMI-1640 and then pressed gently through a 200-mesh metal sieve. The filtrates were lysed with red blood cell lysis buffer in ice for 3 min and centrifuged at 2500 r/min for 3 min to remove the erythrocytes, and the spleen cell suspensions were finally obtained. The spleen cells were suspended in 1 mL of cell culture medium (RPMI-1640 complete medium containing 10% fetal calf serum, 100 U/mL of penicillin, and 0.1 mg/mL of streptomycin), and cell viability was determined by trypan blue staining.

The splenocyte proliferation was assessed by using the CCK8 kit. Briefly, an aliquot of 100 μL of splenocytes suspension (5 × 10^6^ /mL) was seeded in a 96-well plate, then the ConA (Concanavalin A, 100 μL, final concentration 5 μg/mL) were added, 100 μL culture medium RPMI-1640 instead of splenocytes suspension was added in blank contrast group. After incubation at 37°C in an incubator with 5% CO_2_ for 68 h, 20 μL of CCK8 solution was added to each well and incubated for another 4 h. The absorbance at 450 nm was measured on a microplate reader (SMP 500-1780 5-2BF0, Shanghai, China) (Deng et al., 2014). The lymphocyte stimulating index (SI) was calculated by the formula (6):


(6) $$\text{SI}=\frac{OD_{1}}{OD_{2}}$$

where *OD*_1_ was the absorbance value of treatment control, *OD*_2_ was the absorbance value of blank contrast.

### 16S rRNA Amplicon Sequencing

The total microbial DNA was extracted from fecal samples using a DNeasy Power Soil Kit (100) (QIAGEN, Germany). The V3 and V4 regions of the 16S rRNA gene sequence in mice fecal microbiota were determined by high throughput sequencing analysis with the forward primer of 343F (5′-TACGGRAGGCAGCAG-3′), and the reverse primer of 798R (5′- AGGGTATCTAATCCT-3′). The libraries were sequenced on the Illumina MiSeq platform, and the data were analyzed on the free online platform of OE Cloud Platform (Shanghai OE Biotech Co., Ltd, Shanghai, China).

### Statistical Analysis

All statistical analyses were performed by SPSS software (SPSS version 17.0, IBM Institute, United States). The data were expressed as the Mean ± S.D. (standard deviation), and analyzed by one-way analysis of variance (ANOVA) followed by Duncan’s multiple range test to assess the statistical significance, and *P* < 0.05 was considered to be statistically significant.

## Results

### Antioxidant Activities *in vitro* of Protein Hydrolysates and Ultrafiltration Components

**FIGURE 1:**
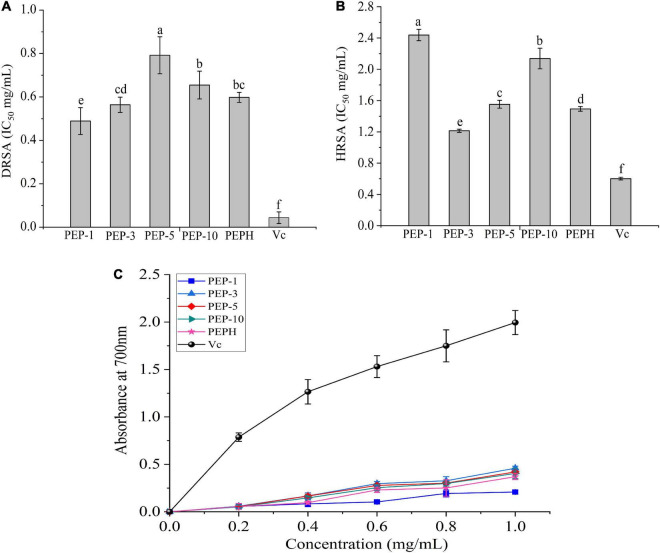
Antioxidant activities *in vitro*. **(A)** DPPH radical scavenging activity expressed as IC_50_, **(B)** hydroxyl radical scavenging activity expressed as IC_50_, and **(C)** reducing power with Vc as the positive control. PEP-1: *P. eryngii* polypeptides with a molecular weight of < 1 kDa; PEP-3: *P. eryngii* polypeptides with a molecular weight of 1–3 kDa; PEP-5: *P. eryngii* polypeptides with a molecular weight of 3–5 kDa; PEP-10: *P. eryngii* polypeptides with a molecular weight of 5–10 kDa; PEPH: PEPH: *P. eryngii* protein hydrolysate; Vc: vitamin C (ascorbic acid).

The antioxidant activities including reducing power, DPPH and hydroxyl radicals scavenging activities were shown in Figure 1. The polypeptides from *P. eryngii* (PEP-1, PEP-3, PEP-5, and PEP-10) showed observable scavenging activities against DPPH and hydroxyl radicals following an increase in the concentrations. The DRSA IC_50_ values of PEPH, PEP-1, PEP-3, PEP-5 and PEP-10 were 0.598 ± 0.023, 0.489 ± 0.062, 0.564 ± 0.035, 0.792 ± 0.085, and 0.655 ± 0.027 mg/mL, higher than these of Vc group (0.044 ± 0.027 mg/mL), respectively. While HRSA IC_50_ values of PEP-3 were lower than those of PEPH markedly, indicating that DPPH radical scavenging activities of PEP-1 were superior to these of PEPH, and PEP-3 have stronger scavenging activities against hydroxyl radicals than PEPH. Moreover, the reducing power increased as the concentration increased from 0 to 1.0 mg/mL (Figure 1C). At a concentration of 1.0 mg/mL, the reducing power of PEP-3 (0.459 ± 0.022) was higher than those of PEPH (0.370 ± 0.031). The assay indicated that ultrafiltration fractions with molecular weight <3 kDa had stronger antioxidant activity, which could add to soybean milk as a functional food additive.

### Amino Acid Analysis of Protein Hydrolysates and Polypeptide With a Small Molecular Weight

A summary of the amino acid composition of protein hydrolysates and polypeptides with a small molecular weight (Mw <3 kDa) was presented in Table 1. Polypeptide with small molecular weight from *P. eryngirr* contained higher concentrations of hydrophobic amino acids (HAA), positively charged amino acids (PCAA), and negatively charged amino acids (NCAA) when compared with protein hydrolysates. The highest contribution to the HAA in a polypeptide with small molecular weight was from Ala, Leu, and Pro, while the concentrations of Asp and Glu played a central role in NCAA.

**TABLE 1:** Amino acid analysis of protein hydrolysates and polypeptide with a small molecular weight.

Amino acids	Protein hydrolysates (g/100g)	Polypeptide with small molecular weight (g/100g)
Aspartic acid (Asp)	5.075 ± 0.34**	7.035 ± 0.15**
Threonine (Thr)	2.276 ± 0.19*	2.467 ± 0.09*
Serine (Ser)	2.194 ± 0.07*	3.448 ± 0.23**
Glutamate (Glu)	13.127 ± 1.19**	14.87 ± 1.11**
Glycine (Gly)	2.407 ± 0.86*	16.764 ± 0.60**
Alanine (Ala)	2.18 ± 0.39*	13.24 ± 0.35**
Cystine (Cys)	0.151 ± 0.01*	0.4 ± 0.05*
Valine (Val)	1.52 ± 0.09*	1.913 ± 0.14*
Methionine (Met)	0.562 ± 0.04*	0.784 ± 0.04*
Isoleucine (Ile)	2.456 ± 0.40*	2.226 ± 0.23
Leucine (Leu)	3.361 ± 0.07*	3.723 ± 0.08**
Tyrosine (Tyr)	2.058 ± 0.39*	2.063 ± 0.91*
Phenylalanine (Phe)	1.975 ± 0.18*	2.491 ± 0.24*
Lysine (Lys)	1.839 ± 0.17*	2.781 ± 0.16**
Histidine (His)	1.225 ± 0.31*	2.192 ± 0.39**
Arginine (Arg)	2.859 ± 0.21*	6.661 ± 0.33**
Proline (Pro)	1.705 ± 0.72*	9.627 ± 0.74**
Hydrophobic amino acids (HAA)	15.966 ± 0.85**	36.465 ± 1.3**
Positively charged amino acids (PCAA)	5.923 ± 0.34**	11.633 ± 0.88**
Negative charged amino acids (NCAA)	18.202 ± 1.53**	21.905 ± 1.26**
Aromatic amino acid (AAA)	4.033 ± 0.56*	4.553 ± 1.15*

Hydrophobic amino acids (HAA): Ala, Val, Ile, Leu, Tyr, Phe, Pro, Met, Cys; Positively charged amino acids (PCAA): Lys, His, Arg; Negative charged amino acids (NCAA): Asp, Glu; Aromatic amino acids (AAA): Phe, Tyr; significant levels: * P < 0.05 and ** P < 0.01.

### Physicochemical Properties of Sour Soybean Milk

The physical and chemical indexes of sour soybean milk fermented with small molecular weight polypeptide (PEP) and protein hydrolysates (PEPH) from *P. eryngii* were shown in Table 2. In brief, the number of lactic acid bacteria in FPEP was significantly increased (*P* < 0.05) when compared with those in SSM, indicating the polypeptides with small molecular weight were beneficial to the growth of lactic acid bacteria. Moreover, the addition of a small molecular weight of polypeptides was good for maintaining a better fermentation flavor, reflecting the decrease of pH and increase of acidity. The WHC of the sour soybean milk fermented with polypeptides (FPEP) was superior to FPEPH and SSM, manifesting that polypeptide could improve the quality and flavor of sour soybean milk.

**TABLE 2:** Physicochemical properties of sour soybean milk.

	SSM	FPEPH	FPEP
Lactic acid bacteria count (× 10^8^ CFU/mL)	1.84 ± 0.10 a	2.26 ± 0.13 b	4.38 ± 0.24 c
pH	4.47 ± 0.07 a	4.30 ± 0.11 ab	4.26 ± 0.09 b
Acidity	57.32 ± 1.06 b	67.35 ± 1.02 a	69.84 ± 2.10 a
WHC(%)	49.75 ± 0.81 b	53.02 ± 0.72 a	53.56 ± 1.30 a

*Means with the same letter are not significantly different (P < 0.05). The same letters indicated no significant differences and different letters indicated significant differences at P < 0.05. WHC: water-holding capacity; SSM: sour soybean milk; FPEP: fermented with P. eryngii polypeptides with a molecular weight of <3 kDa; FPEPH: fermented with P. eryngii protein hydrolysate.*

### Potential Effects in Relieving Immune Organ Atrophy

**FIGURE 2:**
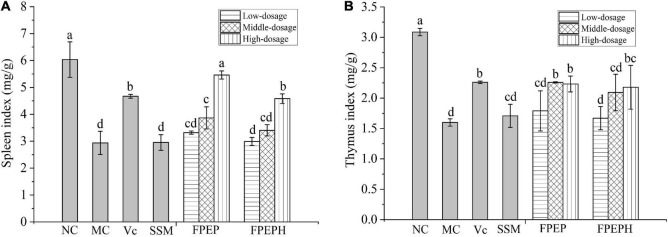
Effects of sour soybean milk on organ index in mice. **(A)** Spleen index, and **(B)** thymus index. Data were presented as the Mean ± S.D. The same letters indicated no significant differences and different letters indicated significant differences at *P* < 0.05. NC: control group; MC: model group; Vc: vitamin C-ascorbic acid; SSM: sour soybean milk; FPEP: fermented with *P. eryngii* polypeptides with a molecular weight of <3 kDa; FPEPH: fermented with *P. eryngii* protein hydrolysate.

The mice in the MC group showed impaired immune symptoms of immune organ atrophy including spleen and thymus. As presented in Figure 2, the spleen and thymus indexes were significantly decreased in the model mice group when compared with those in the NC group, indicating that the immunosuppression mice model induced by CTX was established successfully. However, the mice in the group treated with sour soybean milk showed a conspicuous increase in spleen index than that in the MC group, especially the high-dosage FPEP group, while there was no significant difference in thymus index, manifesting that the sour soybean milk fermented with small molecular weight polypeptides could maintain the normal morphology of immune organs.

### Blood Cell Levels Were Significantly Recovered

**FIGURE 3:**
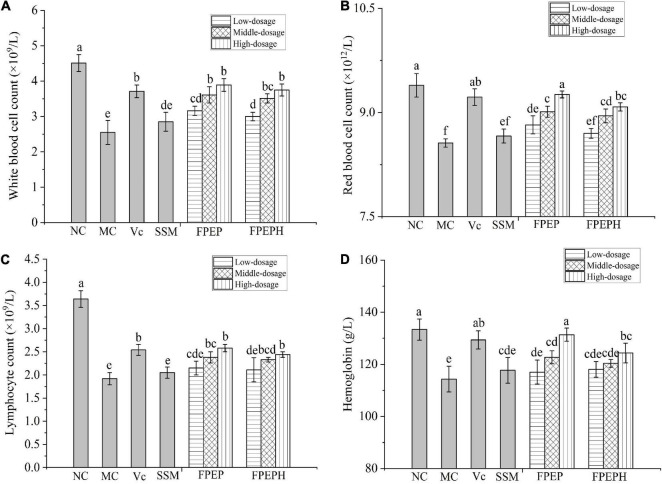
Effects of sour soybean milk on improving blood cells level in mice. **(A)** White blood cell count, **(B)** red blood cell count, **(C)** lymphocyte count, and **(D)** hemoglobin. Data were presented as the Mean ± S.D. The same letters indicated no significant differences and different letters indicated significant differences at *P* < 0.05. NC: control group; MC: model group; Vc: vitamin C-ascorbic acid; SSM: sour soybean milk; FPEP: fermented with *P. eryngii* polypeptides with a molecular weight of <3 kDa; FPEPH: fermented with *P. eryngii* protein hydrolysate.

The levels of blood cells in mice were shown in Figure 3. Specifically, the blood cells count including white blood cells, red blood cells, and lymphocytes were significantly decreased in the MC group induced by CTX when compared to those in the NC group, while the blood cells levels were recovered in the group treated with the sour soybean milk fermented with an extract from *P. eryngii*. The same change trend of hemoglobin was consistent with the blood cells in all groups, indicating that sour soybean milk fermented with polypeptides from *P. eryngii* could inhibit the decrease of blood cells induced by CTX.

### Effects of Sour Soybean Milk on Serum Hemolysin and Splenic Lymphocyte Proliferation

**FIGURE 4:**
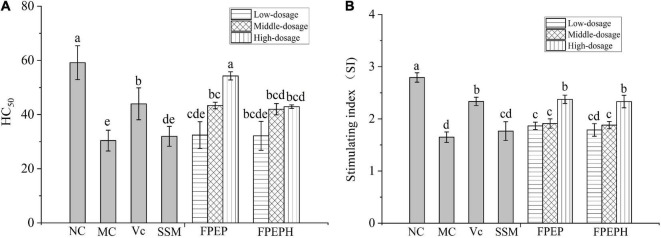
Effects of sour soybean milk on serum hemolysin and splenic lymphocyte proliferation. **(A)** HC_50_, and **(B)** SI. Data were presented as the Mean ± S.D. The same letters indicated no significant differences and different letters indicated significant differences at *P* < 0.05. NC: control group; MC: model group; Vc: vitamin C-ascorbic acid; SSM: sour soybean milk; FPEP: fermented with *P. eryngii* polypeptides with a molecular weight of <3 kDa; FPEPH: fermented with *P. eryngii* protein hydrolysate.

The effects of sour soybean milk on serum hemolysin and splenic lymphocyte proliferation were expressed as HC_50_ and lymphocyte stimulating index (SI) in Figure 4. The HC_50_ values indicated that the levels of serum hemolysin in mice induced by CTX were decreased as a result of the immunosuppression. While the high-dosage FPEP significantly enhanced the levels of serum hemolysin in mice to a level that is statistically comparable to the normal level, suggesting that FPEP could obviously increase the humoral immunity of immunosuppressed mice. Moreover, the proliferation of splenic lymphocytes induced by ConA was used to confirm the effect of sour soybean milk on the cellular immune response. The SI values were shown in Figure 4B, the splenic lymphocyte proliferation in the MC group was significantly lower than that in the NC group, while the SI value of the high-dosage FPEP group was comparable to that of the NC group. These results suggested that sour soybean milk fermented with polypeptides from *P. eryngii* could increase the cellular immunity of immunosuppressed mice.

### Improvement on Antioxidant Activity of Mice

**FIGURE 5:**
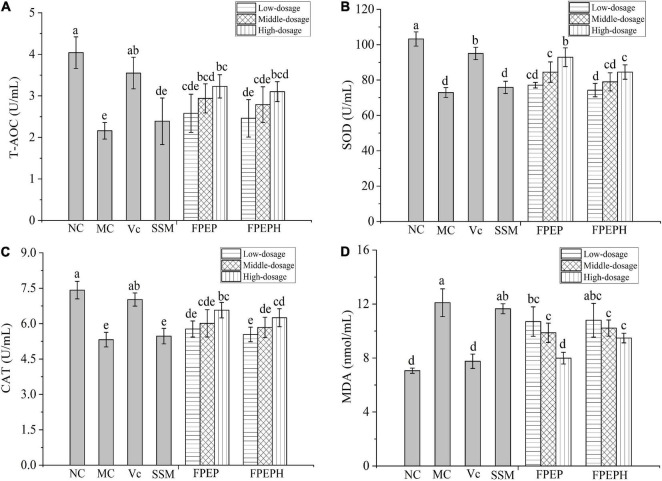
Effects of sour soybean milk on improving antioxidant activity of mice. **(A)** T-AOC, **(B)** SOD, **(C)** CAT, and **(D)** MDA. Data were presented as the Mean ± S.D. The same letters indicated no significant differences and different letters indicated significant differences at *P* < 0.05. NC: control group; MC: model group; Vc: vitamin C-ascorbic acid; SSM: sour soybean milk; FPEP: fermented with *P. eryngii* polypeptides with a molecular weight of <3 kDa; FPEPH: fermented with *P. eryngii* protein hydrolysate; CAT: catalase; MDA: malondialdehyde; SOD: superoxide dismutase; T-AOC: total antioxidant capacity.

As shown in Figure 5, the antioxidant enzyme activities (SOD, CAT, and T-AOC) in the mice of the MC group reduced remarkably, while MDA contents were increased when compared with that in the NC group, manifesting that the balance of oxidation in mice was destroyed by CTX. However, FPEP especially high-dosage FPEP could enhance the enzymatic activity of SOD, CAT, and T-AOC and reduce the content of MDA of the immunosuppressed mice with a dosage-dependent manner.

### Evaluation of Microbial 16S rRNA Gene Sequencing of Intestinal Flora

**FIGURE 6:**
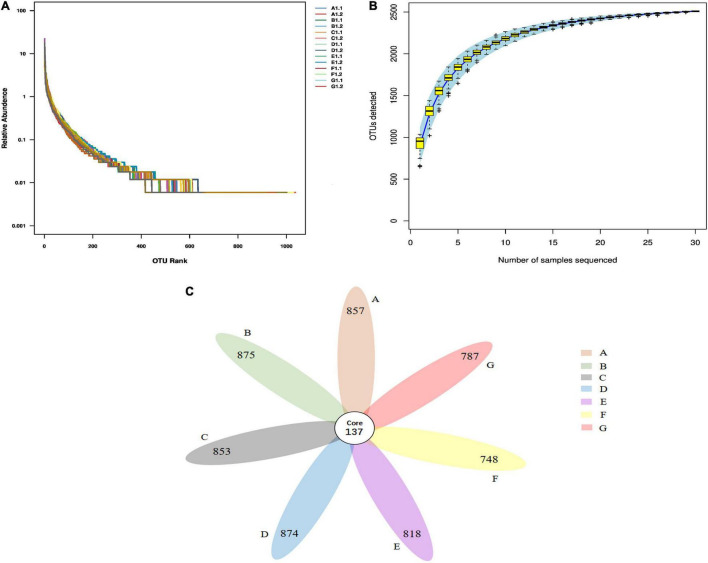
Biodiversity measures of the intestinal microbiota in all of the mice samples. **(A)** OTU rank curve, **(B)** OTU cumulative curve, and **(C)** Petal map of fecal samples in each group. In the petal chart, each petal represents a group, the core number in the middle represents the number of OTUs common to all samples, and the number on the petal represents the number of OTUs unique to the sample. (A: Middle-dosage of FPEP, B: High-dosage of FPEP, C: Middle-dosage of FPEPH, D: High-dosage of FPEPH, E: SSM, F: MC, G: NC). NC: control group; MC: model group; SSM: sour soybean milk; FPEP: fermented with *P. eryngii* polypeptides with a molecular weight of <3 kDa; FPEPH: fermented with *P. eryngii* protein hydrolysate.

Based on the above analysis, FPEP and FPEPH at middle and high dosages showed superior immunological effects, hence, we further evaluated the effects of FPEP or FPEPH with middle and high dosages on the intestinal flora in CTX mice. After gene sequencing and data quality filtering, a total of 702,589 high-quality readings were obtained, and these tags were clustered into OTUs with 97% similarity, and a total of 882-1037 OTUs were obtained. In Figure 6A, the transverse bend line was wider, and the line eventually tends to be flat, indicating that the species composition in the tested samples was abundant and uniform. In Figure 6B, the rising trend at the top of the OTU accumulation curve was gradually stable, suggesting that the sequencing depth in the present study was adequate to cover most of the bacterial diversity in all samples, and following data analysis was feasible. According to the OTU abundance information, petal map analysis was performed to compare the commonness or uniqueness of OTUs between different groups. As shown in Figure 6C, each group had its special community representation. Among them, the high-dosage FPEP and FPEPH groups had more specific OTUs than those of the MC group.

**FIGURE 7:**
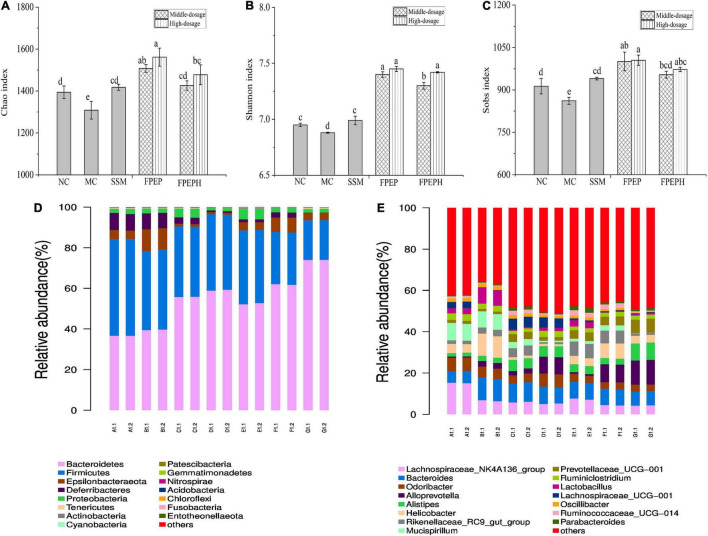
Biodiversity measures, and compositions analysis on phylum and genus levels of the intestinal microbiota in all of the mice samples. **(A–C)** Alpha diversities index of Chao, Shannon, and Sobs, **(D)** Community barplot analysis on phylum level, and **(E)** Community barplot analysis on genus level. (Each group was measured twice in parallel. A: Middle-dosage of FPEP, B: High-dosage of FPEP, C: Middle-dosage of FPEPH, D: High-dosage of FPEPH, E: SSM, F: MC, G: NC). NC: control group; MC: model group; Vc: vitamin C-ascorbic acid; SSM: sour soybean milk; FPEP: fermented with P. eryngii polypeptides with a molecular weight of <3 kDa; FPEPH: fermented with P. eryngii protein hydrolysate; CAT: catalase; MDA: malondialdehyde; SOD: superoxide dismutase; T-AOC: total antioxidant capacity.

The analysis results of alpha diversity were presented in Figure 7. In Figures 7A–C, the observed species index, Chao, Shannon, and Sobs indexes of the PEP and PEPH groups were all higher than that of the MC group, indicating that species richness was lower in the MC group. To better identify the community composition of each sample, the community barplot analysis on phylum and genus levels was carried out. As shown in Figure 7D, *Bacteroidetes* and *Firmicutes* were the two dominant bacteria groups on phylum level in all the samples, followed by *Epsilonbacteraeota*. Observation showed that the relative abundance of *Firmicutes* in the MC group was lower than those in the high-dosage FPEP group, while *Bacteroidetes* in the MC group were higher than those in the high-dosage FPEP group, indicating that the intra-abdominal injection of CTX had a great impact on the two dominant bacteria groups. In Figure 7E, the genera with the highest relative abundance were *Lachnospiraceae_NK4A136_group*, *Bacteroides*, *Helicobacter*, *Odoribacte*, and *Alloprevotella*. The relative abundance of *Bacteroides* in the high-dosage FPEP group was 10.88%, higher than that in the MC group (7.83%). At the same time, the relative abundance of *Lactobacillus* in the MC group was much higher than that in the high-dosage FPEP group. These results indicated that sour soybean milk fermented with polypeptides from *P. eryngii* administration showed effects on enhancing intestinal homeostasis effectively by influencing the bacterial components and abundances.

## Discussion

In recent years, increasing evidence has indicated that peptides derived from mushrooms have been proven to possess excellent effects because of their low molecular weight, and high bioactivity (Bhat et al., 2015). And sour soybean milk has attracted attention in food research due to its health values (Cai et al., 2021). Therefore, sour soybean milk fermented with the polypeptides from *P. eryngii* was obtained successfully and the antioxidant and immunological activities of the mixed sour soybean milk were explored by establishing the immunosuppressed mice induced by CTX in our present work.

Firstly, the protein hydrolysates (PEPH) and ultrafiltration components (PEP-1, PEP-3. PEP-5, and PEP-10) from *P. eryngii* were obtained successfully. And the *in vitro* antioxidant activities were measured to sieve the higher bioactivity polypeptides from *P. eryngii*. Previous literature had reported that antioxidant compounds such as peptides and polysaccharides play a key role in preventing and curing diseases such as cancer, inflammation, and immune disease, and they are important in keeping food quality (He et al., 2013). In the present work, the antioxidant activities of polypeptides with the molecular weight (<3 kDa) were superior to the others, which was consistent with Sun et al. (2017). Moreover, Kimatu et al. (2017) have reported that peptide fractions (<3 kDa) from *Agaricus bisporus* had superior potential bioactive ingredients for use in the formulation of functional foods as well as natural antioxidants in lipid food systems, which was in accord with our results. The antioxidant function of a polypeptide is mostly dependent on its amino acid composition (Liu et al., 2017). The presence of HAA including Ala, Tyr, Met, and Leu has a great contribution to the capacity of antioxidant peptides, and HAA can enhance the solubility of polypeptides in lipids, thereby increasing their interactions with free radicals to achieve antioxidant effects (Xing et al., 2016; Kimatu et al., 2017). The previous report has indicated that NCAA such as Asp and Glu have strong antioxidant activities because they contain more electrons that can be donated to quench free radicals (Nimalaratne et al., 2015). Moreover, Met and Cys could donate its sulfur hydrogen and were considered as an efficient radical scavenger (Zhang et al., 2019). In our present study, the contents of HAA, NCAA, Met, and Cys of the sour soybean milk fermented with PEP were significantly higher than that fermented with PEPH, which was in line with the results of antioxidant activities *in vitro*. Then we have obtained sour soybean milk fermented with small molecular weight polypeptide (PEP) and protein hydrolysates (PEPH) from *P. eryngii* based on the assay of antioxidant activity *in vitro*. The results suggested that the addition of PEP could improve the quality of sour soybean milk including the lower pH, and the higher acidity, WHC, and lactic acid bacteria count, especially the PEP with small molecular weight.

It is well known that CTX is averse to the immune system of the organism and leads to immunosuppression (Zhang et al., 2021), thus we have established the immunosuppression mice model induced by CTX to investigate the immunoregulation effects of sour soybean milk fermented with polypeptides from *P. eryngii*. As the important immune organs, the spleen and thymus indexes could reflect the immune function of the organism. The lower immune organ indexes and the decreased capacity of the immune function in the CTX-treated mice in comparison to normal mice suggested that the immunosuppressed model was established successfully in our present work. While the sour soybean milk fermented with PEP was able to maintain the normal morphology of immune organs and against the immunosuppression.

Moreover, some research demonstrated that hemoglobin might be participating in innate immune responses, and the reduction of red blood cells has a bad influence on the immune-relevant functions of organs (Boisseaux et al., 2017). The change in white blood cell amount can judge immunocompromised diseases, it plays an important role in the defense system of the body (Saba et al., 2018). The lymphocyte, as the important cellular component of the immune response, is the main executor of almost all the immune functions of the lymphatic system (Huang et al., 2021). In our study, the levels of white blood cells, red blood cells, lymphocytes, and hemoglobin were increased by administration with FPEP compared with the MC group, indicating that sour soybean milk fermented with polypeptides from *P. eryngii* could improve the mice immunity. In addition, the serum hemolysin index reflected murine humoral immune function, which is expressed by the HC_50_ values. The higher value of HC_50_ in the FPEP group reflected the positive effects of sour soybean milk fermented with PEP on the complement system of immunosuppressed mice, which was consistent with Deng et al. (2014). Furthermore, the lymphocyte activity was detected through Con A-stimulated spleen lymphocyte proliferation *in vitro*. The results showed that the sour soybean milk fermented with polypeptides could promote the proliferation of spleen lymphocytes in mice, and had the potential effects on enhancing the damaged immune system. Similar conclusions can be obtained by Sun et al. (2017) and Meng et al. (2019).

Furthermore, the incidences of immune diseases could increase once the intestinal symbiotic flora is disturbed. Similar to Tang et al. (2014), our results showed that analysis presented that sour soybean milk fermented with PEP could enhance microbial community diversity by analyzing α-diversity. And *Lactobacillu*s, which belongs to the phylum of *Firmicutes*, has been used as probiotics to regulate immune response by regulating the intestinal flora (Hu et al., 2019). Observation showed that the relative abundances of the phylum of *Firmicutes*, as well as the genus of *Bacteroides* and *Lactobacillus* could increase with the administration of sour soybean milk fermented with polypeptides. All results confirmed the potential effects of sour soybean milk with polypeptides from *P. eryngii* on regulating the microbial community structure and composition, restoring the biological imbalance of intestinal microorganisms in CTX-treated mice. The conclusions were consistent with Ma et al. (2021) and Zhang et al. (2021), which have reported that inflammation-related and immunosuppressed disorders could be regulated by keeping the balance of intestinal flora.

## Conclusion

The current study demonstrated that the qualities of sour soybean milk could improve by adding the small molecular weight polypeptides from *P. eryngii*. Furthermore, sour soybean milk fermented with polypeptide from *P. eryngii* was not only a significant antioxidant, but it also significantly reversed the immunosuppression and maintained the balance of gut microbiota in CTX-treated mice. Thus, the present work provided a reference for studying the potential applications of polypeptides from *P. eryngii* in functional fermented foods. However, further investigations into the mechanism induced by polypeptides and their incorporation into functional foods are needed.

## Data Availability Statement

The data presented in the study are deposited in the NCBI repository, accession number PRJNA804668.

## Ethics Statement

All the experiments were performed in accordance with the Regulations of Experimental Animal Administration issued by the State Committee of Science and Technology of People’s Republic of China, and all mice procedures were reviewed and approved by the Animal Research Ethic Committee of Shandong Agricultural University (Taian, China). Written informed consent was obtained from the individual(s) for the publication of any potentially identifiable images or data included in this article.

## Author Contributions

XS: conceptualization, methodology, data curation, software, and writing—original draft preparation. XX: methodology, data curation, software, and editing. WC: investigation, funding, reviewing, and editing. All authors contributed to the article and approved the submitted version.

## Conflict of Interest

The authors declare that the research was conducted in the absence of any commercial or financial relationships that could be construed as a potential conflict of interest.

## Publisher’s Note

All claims expressed in this article are solely those of the authors and do not necessarily represent those of their affiliated organizations, or those of the publisher, the editors and the reviewers. Any product that may be evaluated in this article, or claim that may be made by its manufacturer, is not guaranteed or endorsed by the publisher.

## Abbreviations

CAT: catalase
CCK-8: Cell counting kit-8
CTX: Cyclophosphamide
DRSA: DPPH radical scavenging activity
FPEP: fermented with PEP (*Pleurotus eryngii* polypeptides with a molecular weight of < 3 kDa)
FPEPH: fermented with PEPH
HAA: hydrophobic amino acids
HRSA: scavenging hydroxyl radicalactivity
MDA: malondialdehyde
Mw: molecular weight
NCAA: negatively charged amino acids
PCAA: positively charged amino acids
PEP: *Pleurotus eryngii* polypeptides
PEPH: *Pleurotus eryngii* protein hydrolysate
SI: stimulating index
SOD: superoxide dismutase
SRBC: sheep red blood cell
SSM: sour soybean milk
T-AOC: total antioxidant capacity
Vc: vitamin C-ascorbic acid
WHC: water-holding capacity.
